# Genome-wide association study for birth, weaning and yearling weight in Colombian Brahman cattle

**DOI:** 10.1590/1678-4685-GMB-2016-0017

**Published:** 2017-05-22

**Authors:** Rodrigo Martínez, Diego Bejarano, Yolanda Gómez, Romain Dasoneville, Ariel Jiménez, Gael Even, Johann Sölkner, Gabor Mészáros

**Affiliations:** 1 Colombian Corporation of Agricultural Research, Tibaitatá Research Center, Bogotá, Colombia; 2 Genes Diffusion, Douai, France; 3 National Association of Zebu 11 Brahman Breeders (ASOCEBU), Bogotá, Colombia; 4 Division of Livestock Sciences, University of Natural Resources and Life Sciences (BOKU), Vienna, Austria

**Keywords:** Bos indicus, SNP, QTL, GWAS, body weight

## Abstract

Genotypic and phenotypic data of 1,562 animals were analyzed to find genomic regions that potentially influence the birth weight (BW), weaning weight at seven months of age (WW) and yearling weight (YW) of Colombian Brahman cattle, with genotyping conducted using Illumina Bead chip array with 74,669 SNPs. A Single Step Genomic BLUP (ssGBLP), approach was used to estimate the proportion of variance explained by each marker. Multiple regions scattered across the genome were found to influence weights at different ages, also dependent on the trait component (direct or maternal). The most interesting regions were connected to previously identified QTLs and genes, such as ADAMTSL3, CAPN2, CAPN2, FABP6, ZEB2 influencing growth and weight traits. The identified regions will contribute to the development and refinement of genomic selection programs for Zebu Brahman cattle in Colombia.

## Introduction

Weight traits are considered the most economically important production traits in beef cattle. The animals are weighed at predefined times to comply with the respective breeding scheme, where common measurements are taken at birth, on weaning and at market age. Apart from quantifying average daily gain, the birth weight is also associated with growth traits in general (Boligon *et al.*, 2009) as well as the mature weight (Meyer, 1995). The weaning weight is used as a criterion to select animals for further breeding (Guidolin *et al.*, 2012). With the availability of dense single nucleotide polymorphism (SNP) genotyping platforms, it is possible to study these traits at the genomic level. The information on regions influencing respective traits could be used as predictors of production potential at an early age, possibly complementing other (genomic) breeding values. Buzanskas *et al.* (2014) identified four regions in connection with birth weight (BTA4 and BTA9), 12 regions for weaning weight (210 days) (BTA4, BTA6 and BTA11) and 10 regions for long-yearling weight (420 days) (BTA7, BTA22, BTA25 and BTA27) in Canchim beef cattle using genome-wide association techniques. A region influencing growth traits was found on BTA14 in Fleckvieh cattle, with follow-up links to calving ease (Pausch *et al.*, 2011). BTA14 was also highlighted in Utsunomiya *et al.* (2013) as the chromosome harboring the most important region for birth weight in Nellore cattle. In crossbred beef cattle, the genomic regions affecting weights of animals at birth, weaning and one year of age were scattered across the genome, with the majority of associations on BTA6, and to a lesser extent on BTA10, BTA11, BTA14 and BTA20 (Snelling *et al.*, 2010).

To our knowledge, a similar examination of genomic regions influencing weight traits is not very common for Brahman cattle. Hawken *et al.* (2012) have reported a GWAS for reproduction traits in two tropically adapted beef cattle breeds, Brahman and Tropical Composite. Bolormaa *et al.* (2011) also performed a GWAS study, but for residual feed intake and growth traits. Additionally they have tested the consistency of SNP effects across different cattle populations and breed types, including the Brahman breed. More recently Crispim *et al.* (2015) have identified associated SNP markers related to growth curves parameters, such as mature weight and maturity rate in Brahman cattle. Studies related to influences of genomic regions controlling weight at specific ages are still scarce though. To our knowledge, a similar examination of genomic regions influencing weight traits is not very common for Brahman cattle. Therefore, the aim of our study was to search for genome-wide associations influencing weight at different ages in Colombian Brahman cattle.

## Material and methods

### Animals and phenotypes

Birth weight (BW), weaning weight (WW) and yearling weight (YW) for 1,234 genotyped animals (475 males and 759 females) from 35 Colombian herds and three regions (North Coast, Oriental Savannas and Andean Valeys) were obtained from the historical databases of the National Association of Zebu Brahman Farmers.

### Genotypes and quality control

The single nucleotide polymorphisms (SNPs) were genotyped using the Bovine Genomic profiler GGPHD 80K BeadChip (GeneSeek, Lincoln, NE), in the Animal Molecular Genetic Laboratory, of GENES DIFFUSION in Lille, France, and at the Molecular Genetic Laboratory of CORPOICA, Colombia, as described previously (Matukumalli *et al.*, 2009). The initial genotype call rates averaged 99.4 ± 0.06% for 74,669 SNPs. In the follow-up procedure, the unplaced SNPs and those on the sex chromosomes were deleted. The remaining SNPs were required to meet the following criteria: call rate minimum 0.90, minor allele frequency minimum 0.05, animal call rate minimum 0.9, and pass parent-progeny test for Mendelian conflicts. After the quality control, a total of 63,971 SNPs and 1,562 genotyped animals remained for the analysis.

### Genome-wide association studies

The analyses were performed using a mixed model and the single-trait model can be expressed as:

y=Xb+Zu+Zm+e

where y is the vector of observations for BW, WW or YW; b is the vector of fixed effects (region, herd, year, season, mating, sex, birth number) u is the vector of random additive genetic effects, combining polygenic breeding values (based on pedigree) and genomic breeding values (based on genotypes) and m is the vector of maternal genetic effects; X and Z are incidence matrixes; e is the vector of random residuals. For genomic analysis, an ssGBLUP (Misztal *et al.*, 2009; Aguilar *et al.*, 2010) was used. This method integrates the genomically derived relationship matrix (G) with population-based pedigree relationships matrix (A) into a combined relationship matrix (H) as was described by Legarra *et al.* (2009) and allows for genomic selection in a single step.

For SNPs estimation, the windows variance option was used to compute the proportion of variance explained by each window of 4 SNP markers, as was described by Fragomeni *et al.* (2014). The regions were defined as the positions of the SNPs with the highest variance explained ± 0.5 Mb in each direction. The genomic analysis and SNP identification were based on the Bos_taurus_UMD_3.1.1 assembly of the bovine genome sequence (UMD_3.1.1/bosTau8). To provide information regarding the identity and function of genes at mapped SNP markers, the chromosomal positions at the Ensembl Genome Browser were used (http://www.ensembl.org/index.html). Lists of genes located nearest to the significant SNP were extracted, allowing for a maximum distance of 1 Mb between SNP and annotated genes.

## Results and Discussion

**Figure 1:**
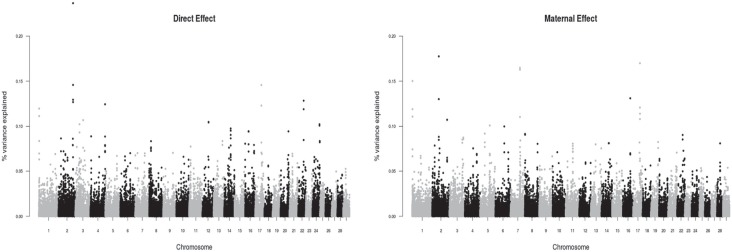
Manhattan plot for direct and maternal effects for birth weight in Zebu Brahman cattle in Colombia.

**Figure 2:**
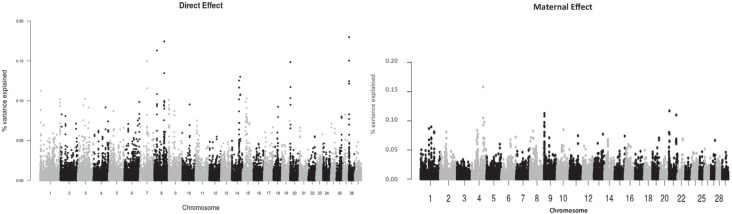
Manhattan plot for direct and maternal effects for weaning weight at 7 months in Zebu Brahman cattle in Colombia.

**Figure 3:**
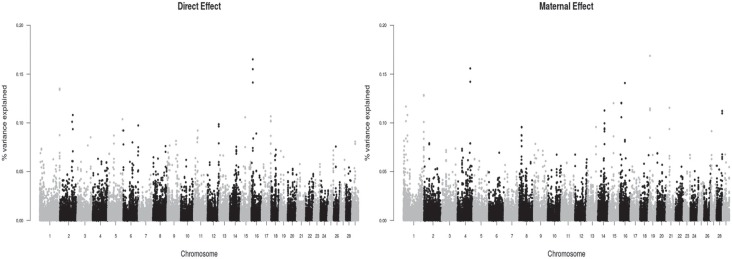
Manhattan plot for direct and maternal effects for yearling weight at 12 months in Zebu Brahman cattle in Colombia.

The regions explaining a large proportion of trait variation influencing BW, WW or YW in Colombian Brahman cattle were identified based on the effect for each SNP. Both direct and maternal components were used to identify the region important for both, as well as any differences between them. Table 1 displays the descriptive statistics for each trait and heritability values ranging between 0.18 to 0.26, indicating moderate magnitude of the direct genetic effect for growth traits in Brahman cattle. The phenotypic correlations between traits were high and positive, ranging between *R* ^2^ = 0.93 for WW and YW, and *R* ^2^ = 0.97 for BW and WW. The results for the whole genome are shown in Manhattan plots in Figures 1–3. The genomic regions are more precisely identified in Tables 2–4, including the number of characterized protein-coding genes in the region (NCBI, annotation release 104) and any growth-related QTLs (CattleQTLdb, Release 27).

**Table 1:** Descriptive statistics for growth, reproductive and ultrasound traits in the Zebu Brahman population.

Traits	Mean	S. D.	C. V.	Min	Max	h2_d_
BW	33.06	3.60	10.89	19	68	0.26
WW	198.31	25.99	13.11	143	266	0.18
YW	257.46	25.47	9.89	205	340	0.22

h2_d_: Direct heritability, S.D.: standard deviation, C.V. Coefficient of variation

BW: Birth weight, WW: weaning weight, YW: Yearling weight

**Table 2:** Most important regions of DNA related to birth weight in Zebu Brahman breed.

Trait type	Chromosome	Region^a^	Number of characterized genes in the region^b^	Weight and growth-relevant QTLs
Direct and maternal	BTA2	17.34 −18.42	5	QTL:1549 c, “Body depth”
				QTL:1550, “Body form composite index”
				QTL:3491, “Hip height”
				QTL:1553, “Stature”
				QTL:1323, “Body weight (birth)”
		51.52 – 52.55	1	QTL:10670, “Body weight (birth)”
				QTL:10665, “Carcass weight”
				QTL:1310, “Body weight (slaughter)”
	BTA17	49.40 – 53.40	5	QTL:23867, “Metabolic body weight”
				QTL:23868, “Metabolic body weight”
				QTL:23870, “Body weight (weaning)”
Direct only	BTA16	27.83 – 28.86	9	QTL:11025, “Body weight (birth)”
				QTL:11026, “Body weight (weaning)”
				QTL:4482, “Body weight (weaning)”
				QTL:4486, “Pre-weaning average daily gain”
Maternal only	BTA1	1.62 – 2.65	9	QTL:24744, “Weaning weight-maternal milk”
				QTL:5265, “Residual feed intake”
	BTA7	73.22 – 74.28	10	No weight and growth-related QTL
	BTA16	58.20 – 60.56	6	No weight and growth-related QTL

a The region is defined as the location of the most important SNPs ± 0.5 Mb

b Source: NCBI, October 2015, annotation release 104

c QTL number identification on QTLdatabase (www.animalgenome.org/cgi-bin/)

**Table 3:** Most important regions of DNA related to weaning weight in Zebu Brahman breed.

Trait type	Chromosome	Region^a^	Number of characterized genes in the region^b^	Weight and growth-relevant QTLs
Direct and maternal	BTA7	56.96 – 57.99	2	QTL:10807^c^, “Body weight (yearling)”
				QTL:10806, “Longissimus muscle area”
				QTL:10805, “Carcass weight”
				QTL:10808, “Body weight (weaning)”
				QTL:10810, “Body weight (birth)”
	BTA8	1.63 – 2.65	2	QTL:10821, “Body weight (weaning)”
				QTL:10820, “Height (mature)”
				QTL:10819, “Longissimus muscle area”
				QTL:10817, “Carcass weight”
		78.35 – 79.40	7	No matching QTLs
	BTA14	57.26 – 58.34	4	No QTLs
	BTA20	2.70 – 3.74	6	QTL:1357, “Body weight (birth)”
				QTL:11099, “Body weight (birth)”
				QTL:11098, “Carcass weight”
				QTL:11097, “Body weight (weaning)”
				QTL:11094, “Carcass weight”
				QTL:11096, “Body weight (yearling)”
	BTA28	1.91 – 2.97	4	No QTLs

a The region is defined as the location of the most important SNPs ± 0.5 Mb

b Source: NCBI, October 2015, annotation release 104

c QTL number identification on QTLdatabase (www.animalgenome.org/cgi-bin/)

**Table 4:** Most important regions of DNA related to yearling weight in Zebu Brahman breed.

Trait type	Chromosome	Region^a^	Number of characterized genes in the region^b^	Weight and growth-relevant QTLs
Direct and maternal	BTA1	155.24 - 156.42	4	No matching QTLs
	BTA16	11.35 – 12.42	0	QTL:11019 c, “Body weight (yearling)”
				QTL:11022, “Carcass weight”
				QTL:11021, “Longissimus muscle area”
				QTL:11023, “Weaning weight-maternal milk”
				QTL:1355, “Carcass weight”
				TL:23153, “Body weight (weaning)”
	BTA19	9.39 – 10.49	17	QTL:11075, “Body weight (mature)”
				QTL:11076, “Body weight (yearling)”
Maternal only	BTA1	17.76 - 18.82	3	QTL:10639, “Weaning weight-maternal milk”
				QTL:10638, “Body weight (birth)”
	BTA14	51.05 – 52.15	1	QTL:23842, “Body weight (weaning)”
				QTL:23846, “Body weight (weaning)”
				QTL:23843, “Body weight (birth)”
				QTL:23844, “Body weight (birth)”
				QTL:23845, “Body weight (birth)”
				QTL:23265, “Carcass weight”
				QTL:23849, “Metabolic body weight”
				QTL:23850, “Metabolic body weight”
	BTA21	23.48 - 24.62	10	QTL:22801, “Average daily gain”
				QTL:11118, “Fat thickness at the 12th rib”
				QTL:2641, “Body weight (birth)”
				QTL:11123, “Body weight (mature)”
				QTL:11122, “Height (mature)”
				QTL:11120, “Height (yearling)”
	BTA28	42.28 – 43.47	11	QTL:22763, “Body weight (birth)”

a The region is defined as the location of the most important SNPs ± 0.5 Mb

b Source: NCBI, October 2015, annotation release 104

c QTL number identification on QTLdatabase (www.animalgenome.org/cgi-bin/)

### Birth weight

For BW, the highest SNP effects were found on BTA2 and BTA17, for maternal and direct effects, on BTA16 for the direct effect only, and on BTA1, BTA7 and BTA16 for the maternal effect only (Figure 1). Most of the regions were harboring QTLs connected with growth and development, although not all of those were directly linked to BW. In addition, multiple genes were located in the identified regions (Table 2), although only a few could be connected to growth directly. The most important genes were the zinc finger E-box binding homeobox 2 (ZEB2) (Yamakoshi *et al.*, 2012) on chromosome 2. This gene is involved in pathways regulating early growth and development, fatty acid binding protein 6, (Zheng *et al.*, 2009), skeletal muscle growth rates and with calpain 2 (CAPN2) which is associated with growth in cattle (Zhang and Li, 2011).

### Weaning weight

For this trait the regions explaining a large proportion of trait variation were different for direct and maternal effects. The regions with the highest variance explained for direct effect were found on BTA7, BTA8, BTA14, BTA20 and BTA28 (Figure 2). The identified locations matched known QTL regions for growth on BTA7, BTA8 and BTA20, but not in the other chromosomes. In addition there were QTLs for milk production in almost all identified regions. These QTLs might be connected to growth rate in the pre-weaning period, as the portion of the diet for young calves is based on milk. In addition to QTLs there were a number of SNPs characterized in our regions, but none of them with an apparent strong link to the growth rate in cattle or other organism (Table 3).

The regions with the highest variance explained for maternal effect were on BTA4, BTA9 and BTA21, with QTLs for milk production, residual feed intake and body weight. Two regions on BTA4, the first one located around 93.9 Mb with four SNPs with the highest effect. The second one located around 44.3 to 44.7 Mb contained important QTLs at 44.7-44.9 Mb reported by Collis *et al.* (2012). The LEPT gene has been mapped at 93.24 to 93.26 Mb. Woronuk *et al.* (2012) reported that animal weight was also shown to be positively associated (p < 0.0001) with a single nucleotide polymorphism (C/T) in bovine leptin, located at the same position detected in this study. Two different significant regions have been found on BTA21, the first around 4.1 Mb and the second located around 63.0 Mb, including three QTLs related to growth in cattle (Lu *et al.*, 2013b). Finally, on BTA9 the region with 11 SNPs contained two QTLs related to milk fat yield and the longissimus muscle area.

### Yearling weight

For YW, we found signals on BTA1, BTA16 and BTA19 for direct and maternal effects, and on BTA1, BTA4, BTA 14, BTA21 and BTA28 for maternal effects only, all controlling between 0.10 to 0.18 of the variance (Figure 3). Again, most regions were harboring QTLs connected to growth. Interestingly, in one of the regions explaining a large proportion of trait variation (BTA1, 155-156Mb), the only QTL was for “shear force”. In addition, none of the four characterized genes had any apparent connection to growth (Table 4). Upon closer examination, we found the calpain 7 (CAPN7) gene (BTA1, 154 Mb), which was slightly out of our primary region, but is know for its significant influence on growth in farm animals (Yang *et al.*, 2009). Another interesting gene was the ADAMTS-like 3 (ADAMTSL3) on BTA21, with confirmed connections to body size and growth in cattle (Liu *et al.*, 2012) and humans (Lettre *et al.*, 2008).

In our study, we have used a single-step genome-wide association approach to identify regions connected to birth, weaning and yearling weight in Colombian Brahman cattle. The advantage of the method is that it uses phenotypes from both genotyped and non-genotyped individuals by blending relationship matrixes (Wang *et al.*, 2012, 2014). The regions with the highest proportion of variance explained for the respective trait were matching for WW, with some private regions of interest for BW and YW in respect to direct and maternal effects.

The regions denoted by SNPs with the highest variance explained were scattered across the genome (Figures 1–3). Interestingly, none of these regions was identified in more than one trait. While some of the identified regions were on the same chromosome across traits (*e.g.* on BTA16 for birth and yearling weight), these were far enough from each other to distinguish them empirically as separate signals. In general, the identified regions corresponded to locations of previously identified QTLs related to growth and weight traits. The most interesting exception was the 57-58 Mb region on BTA14 harboring no QTLs at all, despite an apparently rich QTL region downstream (BTA14: 51-52Mb) identified for yearling weights.

We have identified multiple genes in our regions, such as ADAMTSL3, CAPN2, CAPN2, FABP6, ZEB2 influencing growth and weight traits in cattle, or their homologous forms in other organisms. Contrary to the studies on Brazilian Nellore cattle (Utsunomiya *et al.*, 2013) the region harboring PLAG1 on BTA14 was not associated with any of the traits, this gene also was reported by Karim *et al.* (2011) but influencing bovine stature. Additional associations were reported on BTA9 for birth weight and BTA6, BTA11 for weaning weight, and BTA7, BTA22 and BTA27 for yearling weight (Lu *et al.*, 2013a; Buzanskas *et al.*, 2014). None of these regions corresponded to our study. Thus, our findings warrant further investigations to search for common regions or to identify unique regions influencing weight traits in Colombian Brahman cattle.

## Conclusion

In this study we used a single-step genome-wide association approach to identify the regions connected to birth, weaning and yearling weight in Colombian Brahman cattle. The regions with the highest proportion of variance explained (including a ± 0.5Mb buffer region) were considered as candidates influencing the weight traits. Signals were scattered across the genome, connected to previously identified QTL regions and genes such as ADAMTSL3, CAPN2, CAPN2, FABP6, ZEB2 influencing growth and weight traits. Similar regions were responsible for controlling birth, weaning and yearling weights, when direct and maternal effects were considered, but there was no overlap between traits. These results confirm that multiple different regions influence the weight traits, which should be considered during the development and refinement of genomic selection programs for Zebu Brahman cattle in Colombia.
